# Association of serum oleic acid level with depression in American adults: a cross-sectional study

**DOI:** 10.1186/s12888-023-05271-0

**Published:** 2023-11-16

**Authors:** Jiahui Yin, Siyuan Li, Jinling Li, Rongpeng Gong, Zhixia Jia, Junjun Liu, Zhi Jin, Jiguo Yang, Yuanxiang Liu

**Affiliations:** 1 College of Traditional Chinese Medicine, Shandong University of Traditional Chinese Medicine, Jinan, China; 2 Department of Psychiatry, Shanghai Mental Health Center, Shanghai Jiao Tong University School of Medicine, Shanghai, China; 3 College of Acupuncture and Massage, Shandong University of Traditional Chinese Medicine, Jinan, China; 4 RWTH Aachen University, Aachen, Germany; 5 First Clinical Medical College, Shandong University of Traditional Chinese Medicine, Jinan, China; 6 Nanjing Meishan Hospital, Nanjing, China; 7 Department of Neurology, Shanghai Fifth People’s Hospital, Fudan University, Shanghai, China; 8 Department of Neurology, Shandong University of Traditional Chinese Medicine Affiliated Hospital, Jinan, China

**Keywords:** Depression, Oleic acid, Fatty acid, Cross-sectional study

## Abstract

**Background:**

As the most abundant fatty acid in plasma, oleic acid has been found to be associated with multiple neurological diseases; however, results from studies of the relationship between oleic acid and depression are inconsistent.

**Methods:**

This cross-sectional study analyzed 4,459 adults from the National Health and Nutrition Examination Survey 2011–2014. The following covariates were adjusted in multivariable logistic regression models: age, sex, race/ethnicity, education level, marital status, body mass index, physical activity, smoking status, alcohol status, metabolic syndrome, omega-3 polyunsaturated fatty acids, and total cholesterol.

**Results:**

Serum oleic acid levels were positively associated with depression. After adjusting for all covariates, for every 1 mmol/L increase in oleic acid levels, the prevalence of depression increased by 40% (unadjusted OR: 1.35, 95%CI: 1.16–1.57; adjusted OR: 1.40, 95% CI: 1.03–1.90).

**Conclusions:**

Our study suggests that oleic acid may play a role in depression. Further research is needed to investigate the potential benefits of changing oleic acid levels for the treatment and prevention of depression.

**Supplementary Information:**

The online version contains supplementary material available at 10.1186/s12888-023-05271-0.

## Introduction

Depression is a common psychological symptom associated with clinical depressive disorders such as major depressive disorder (MDD). More than 300 million people worldwide live with MDD, of whom 15% (48.16 million) are in the Americas [1]. During the coronavirus disease-19 pandemic, depression levels have risen dramatically [2, 3]. Depression is associated with various physical diseases and higher mortality, adversely affecting patients’ quality of life [4]. Therefore, effective preventive techniques and modifiable risk factors for depression should be investigated.

Fatty acids (FAs) are important compounds involved in various physiological processes, such as cell and tissue metabolism, function, and responsiveness to hormonal and other signals [5]. Several studies have demonstrated that FAs alter neurotransmission, cell survival, and neuroinflammation, which may play a role in neurological diseases [6]. Recent research has mostly focused on polyunsaturated fatty acids (PUFAs), whereas few studies have examined the relationship between plasma omega-9 monounsaturated fatty acids (MUFAs) and depression. Oleic acid, the most abundant FA in plasma, accounts for approximately 80% of plasma phospholipid MUFAs [7].

Previous studies have shown changes in oleic acid levels in patients with MDD; however, the results have been inconsistent. Ding et al. And Zhou et al. observed a decrease in oleic acid levels in patients with MDD [8, 9]. However, McNamara et al. found no significant differences in oleic acid levels between patients with MDD and healthy controls in the superior temporal gyrus [10]. Interestingly, in a case–control study of recurrent MDD, plasma oleic acid levels were significantly higher in patients with recurrent MDD than in the healthy controls [11].

Oleic acid intake has also been linked to anxiety and depression in women [12, 13]. In addition, the rate-limiting enzyme of oleic acid, stearoyl-CoA desaturase (SCD), has been found to cause neurotoxicity by producing MUFA and impairing microglia and macrophages [14]; it also plays a role in the pathogenesis of various diseases, such as obesity, Parkinson’s disease, and Alzheimer’s disease [15–17]. However, few studies have explored the relationship between circulating oleic acid levels and depression, and the role of oleic acid in depression remains unknown.

Therefore, we aimed to explore the relationship between serum oleic acid levels and depression in a multi-ethnic community from the National Health and Nutrition Examination Survey (NHANES).

## Methods

### Study population

The participants in this study were recruited from the NHANES, a major program conducted by the Centers for Disease Control and Prevention (CDC) to assess the health and nutritional status of adults and children in the United States of America. The NHANES comprises demographic, socioeconomic, health-related, and medical information. NHANES survey is approved by the research ethics review board of the Centers for Disease Control and Prevention [18]. All participants provided informed consent. The NHANES data are publicly available. There was no additional authorization or ethical review required for the release of NHANES data for this study. Further information regarding the NHANES is available on the CDC website [19].

Participants in our study were screened on the basis of the following inclusion criteria: 1) age 18 years or older, 2) participation in serum fatty acid testing and availability of fasting laboratory specimens. The exclusion criteria were as follows: 1) incomplete Patient Health Questionnaire-9 (PHQ-9) (*n* = 452) and 2) missing information on oleic acid level (*n* = 46).

### Measurement of serum oleic acid levels

Esterified fatty acids are hydrolyzed primarily from triglycerides, phospholipids and cholesteryl esters using sequential treatment with mineral acid and base in the presence of heat. Using a modification of [20], total fatty acids are hexane-extracted from the matrix (100uL serum) along with an internal standard solution containing stable isotopically-labeled fatty acids to account for recovery. The extract is derivatized with pentafluorobenzyl bromide (PFBBr) in the presence of triethylamine to form pentafluorobenzyl esters. The reaction mixture is injected onto a capillary gas chromatograph column to resolve oleic acid from other matrix constituents. Oleic acid is detected using electron capture negative-ion mass spectrometry. Oleic acid is measured using selected ion monitoring. Quantitation is accomplished by comparing the peak area of the analyte in the unknown with the peak area of a known amount in a calibrator solution. Calculations are corrected based on the peak area of the internal standard in the unknown compared with the peak area of the internal standard in the calibrator solution. Serum samples were processed, stored, and shipped to the Division of Laboratory Sciences, National Center for Environmental Health, CDC, Atlanta, GA, for testing. More information regarding laboratory techniques and quality assurance has been previously documented [21].

### Assessment of depression

The 9-item PHQ-9 was used to assess depression. The PHQ-9 is well accepted as an accurate and reliable technique for depression screening [22–24]. The PHQ-9 contains nine items regarding the frequency of depressive symptoms [25]. Each question is scored from “0” (not at all) to “3” (nearly every day). The total PHQ-9 score can vary from 0 to 27, and a score of ≥ 10 was defined as depression in this study. Based on the cutoff point, the PHQ-9 achieved a sensitivity of 85% and a specificity of 89% for detecting MDD [26].

### Covariates

Model covariates were selected based on a priori knowledge [11, 27]. Self-reported sociodemographic characteristics included age, sex (male/female), race/ethnicity (non-Hispanic white, Mexican American, non-Hispanic black, other Hispanic, or other race/multiple races), education level (< high school/completed high school or > high school), and marital status (married/living with partner or never married/widowed/divorced/separated).

Physical activity was assessed by vigorous physical activity (high-intensity activities and fitness and sports such as running or basketball) and moderate physical activity (e.g., brisk walking, swimming, and bicycling at a regular pace) reported by participants. Alcohol status and smoking status were used as categorical variables. Body mass index (BMI) was measured as weight (kg) divided by height (m) squared. Alcohol status was determined by answers to the question, “Have you had at least 12 alcohol drinks a year?” (yes/no). Participants were divided into never smokers (smoked < 100 cigarettes), former smokers (currently not smoking but smoked ≥ 100 cigarettes), and current smokers (≥ 100 cigarettes and currently smoking every day or some days).

Metabolic syndrome (MetS) was defined according to the updated National Cholesterol Education Program/Adult Treatment Panel III criteria for Americans [28].

Omega-3 PUFA levels were detected using electron capture negative-ion mass spectrometry. Total cholesterol levels were measured using the enzymatic method, which is a single-reagent, endpoint reaction specific for cholesterol.

We have collated the above confounding variables and their detailed definitions into Table S1.

### Process of extracting data

First we searched the NHANES database for oleic acid data and PHQ-9 questionnaire data. We found that this information was collected in the 2011–2014 surveys. Next, covariate information was retrieved for participants in the 2011–2014 surveys, and the download websites for the covariates were identified. We downloaded the required data files locally and combined the data according to the unique identification number assigned to each participant by NHANES. Then, we arrange and clean the combined data to obtain the final dataset to be analysed.

### Statistical analyses

Participant characteristics are expressed as means (95% CIs) for continuous variables and as proportions and percentages of the total for categorical variables. Continuous data were compared using one-way analysis of variance, and categorical data were compared using the chi-squared test. Restricted cubic spline (RCS) analysis with 5 knots (5th, 28th, 50th, 73th, and 95th percentiles) was used to characterize the shape of the association between oleic acid levels and depression. Multivariable logistic regression analysis was performed to quantify the association between oleic acid levels and depression. We used three levels of adjustment: Model 1 was adjusted for age, sex, and race/ethnicity; Model 2 was further adjusted for education level, marital status, physical activity, BMI, smoking status, and alcohol status; and Model 3 was additionally adjusted for MetS, omega-3 PUFAs, and total cholesterol. We also performed a sensitivity analysis to ensure that the results were robust. Oleic acid was converted into a categorical variable (quartiles), and the *p*-value for trend was calculated. This method provides a more visual representation of the changes of depression with increasing oleic acid levels [29–32].

In total, 456 (10.2%) participants were excluded from the analyses because of missing values in their covariates. These participants were not included in the analyses that adjusted for the corresponding missing covariates. Imputations of missing data were conducted for sensitivity analyses using the missForest R package, which is a random forest-based technique that is highly computationally efficient for high-dimensional data consisting of both categorical and continuous predictors [33]. Subgroup analyses were performed using stratified multivariable logistic regression analyses.

All statistical analyses in this study were conducted in accordance with CDC guidelines. All analyses were performed using R (The R Foundation, Vienna, Austria) and Empower (X & Y Solutions, Boston, MA). A two-sided *p*-value < 0.05 was considered statistically significant.

## Results

### Participant characteristics

In total, 4,459 participants were included for statistical analysis (Fig. S1). Table 1 shows the characteristics of participants according to quartiles of serum oleic acid levels (Q1, ≤ 1.54 mmol/L; Q2, > 1.54 mmol/L to 1.94 mmol/L; Q3, > 1.94 mmol/L to < 2.51 mmol/L; Q4, ≥ 2.51 mmol/L). Participants with the highest oleic acid level (Q4; ≥ 2.51 mmol/L) were more likely to be older, non-Hispanic White, married or living with the partner, and a current or former smoker; have lower physical activity; have higher BMI values, omega-3 PUFA levels, and total cholesterol levels; and were more likely to have MetS and depression than participants in the other groups (all *p*-value < 0.05).

**Table 1:** Characteristics of study participants aged ≥ 18 years from the 2011–2014 National Health and Nutrition Examination Survey by oleic acid level (*n* = 4,459)

Characteristic	Overall	Oleic acid (18:1n-9) quartiles, mmol/L				*p*-value
		Q1	Q2	Q3	Q4	
		(≤ 1.54 mmol/L)	(> 1.54 to 1.94 mmol/L)	(> 1.94 to < 2.51 mmol/L)	(≥ 2.51 mmol/L)	
Oleic acid level, mmol/L, mean (95% CI)	2.21 (2.14, 2.29)	1.29 (1.27, 1.31)	1.74 (1.73, 1.75)	2.19 (2.18, 2.20)	3.49 (3.35, 3.63)	
Sample size, *n* (%)	4459 (100)	1136 (25.48)	1105 (24.78)	1103 (24.74)	1115 (25.01)	
Male, *n* (%)	2207 (49.5)	518 (46.84)	532 (47.39)	547 (47.33)	610 (55.37)	0.013
Age, years, mean (95% CI)	46.53 (45.59, 47.47)	39.05 (37.51, 40.60)	46.30 (44.66, 47.95)	48.82 (47.43, 50.21)	51.14 (49.92, 52.37)	< 0.001
Educational level, *n* (%)						0.409
< High school	1015 (22.77)	236 (16.24)	241 (17.02)	265 (18.45)	273 (16.98)	
Completed high school	964 (21.63)	264 (23.21)	239 (20.28)	223 (17.85)	238 (21.26)	
> High school	2478 (55.6)	636 (60.56)	624 (62.70)	615 (63.71)	603 (61.76)	
Race/ethnicity, *n* (%)						< 0.001
Non-Hispanic White	1880 (42.16)	364 (55.28)	471 (68.66)	494 (71.10)	551 (73.19)	
Non-Hispanic Black	948 (21.26)	394 (21.48)	256 (12.38)	178 (7.52)	120 (5.28)	
Mexican American	539 (12.09)	105 (8.25)	116 (7.08)	166 (9.21)	152 (8.43)	
Other Hispanic	436 (9.78)	103 (6.55)	96 (5.00)	118 (6.26)	119 (5.73)	
Other race/multiple races	656 (14.71)	170 (8.44)	166 (6.88)	147 (5.92)	173 (7.36)	
Marital status, *n* (%)						0.044
Married/Living with partner	2497 (59.3)	524 (57.22)	649 (65.02)	651 (65.88)	673 (64.00)	
Widowed/Divorced/Separated/Never married	1714 (40.7)	477 (42.78)	405 (34.98)	413 (34.12)	419 (36.00)	
Alcohol status, *n* (%)	3197 (71.79)	809 (76.44)	775 (76.05)	787 (78.27)	826 (80.42)	0.162
Smoking status, *n* (%)						< 0.001
Never smoking	2501 (57.64)	693 (63.70)	646 (59.54)	613 (56.48)	549 (49.83)	
Former smoker	1000 (23.05)	211 (19.47)	252 (24.16)	249 (24.09)	288 (26.57)	
Current smoker	838 (19.31)	176 (16.83)	179 (16.30)	218 (19.43)	265 (23.59)	
Physical activity,* n* (%)						< 0.001
Inactive	2178 (48.85)	492 (42.43)	532 (45.18)	545 (46.12)	609 (52.50)	
Moderate	1224 (27.45)	270 (22.43)	321 (30.42)	323 (30.06)	310 (29.68)	
Vigorous	400 (8.97)	154 (13.56)	89 (8.36)	86 (7.46)	71 (6.29)	
Both moderate and vigorous	657 (14.73)	220 (21.57)	163 (16.05)	149 (16.35)	125 (11.52)	
BMI, n (%)						< 0.001
< 25.0 kg/m2	1391 (31.48)	460 (41.76)	382 (34.50)	320 (29.68)	229 (17.89)	
25.0 to < 30.0 kg/m2	1423 (32.21)	342 (31.67)	356 (33.46)	355 (33.52)	370 (33.46)	
≥ 30.0 kg/m2	1604 (36.31)	330 (26.56)	356 (32.04)	418 (36.80)	500 (48.65)	
Total cholesterol, mmol/L, mean (95% CI)	4.93 (4.88, 4.97)	4.27 (4.20, 4.34)	4.69 (4.64, 4.75)	5.13 (5.05, 5.21)	5.53 (5.45, 5.61)	< 0.001
Omega-3 PUFAs, μmol/L, mean (95% CI)	363.63 (353.97, 373.29)	269.95 (259.92, 279.98)	320.08 (305.85, 334.31)	366.08 (353.66, 378.50)	485.11 (473.09, 497.13)	< 0.001
Metabolic Syndrome, *n* (%)	1463 (33.09)	173 (12.81)	245 (20.95)	348 (28.39)	697 (65.04)	< 0.001
Depression, *n* (%)	383 (8.59)	66 (5.69)	87 (6.87)	94 (7.23)	136 (11.19)	0.002

*Abbreviations*: *NHANES* National Health and Nutrition Examination Survey, *Q* Quantile, *BMI* Body mass index, *PUFAs* Polyunsaturated fatty acids

A comparison of characteristics between participants included and excluded (excluded for the absence of oleic acid level data or incomplete PHQ-9) from the analysis is shown in Table S1 in the Supplementary Material. Missing data are listed in Table S2. To avoid the effect of outliers on the results, we excluded outlier patients (1.05%) from subsequent analysis. Owing to the skewed distribution of serum oleic acid levels, outliers were identified using the Huber method [34].

### Association of oleic acid level and depression

**Fig. 1:**
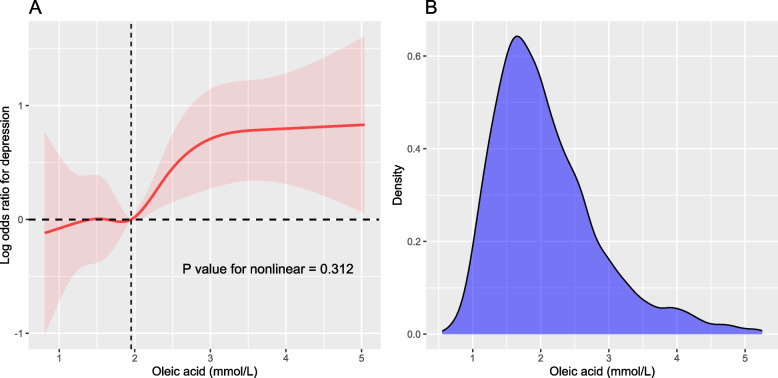
Restricted cubic spline of the relationship between serum oleic acid levels and depression. Adjusted for age, sex, race/ethnicity, education level, marital status, body mass index, physical activity, smoking status, alcohol status, metabolic syndrome, omega-3 polyunsaturated fatty acids, and total cholesterol (*n* = 3928). In (**A**), the solid line represents the line of best-fit, and the pale pink area represents the 95% confidence interval. **B** is density curves of serum oleic acid levels

According to RCS, oleic acid levels were positively associated with depression (Fig. 1). Using multivariable logistic regression analysis, Table 2 quantifies the relationship between oleic acid levels and depression. Oleic acid was positively linked with depression in the crude model (OR = 1.35, 95% confidence interval [CI]: 1.16–1.57, *p* = 0.003). A significant correlation between oleic acid levels and depression was still detected in models after accounting for various variables (model 1: OR = 1.40, 95% CI: 1.21–1.61; model 2: OR = 1.29, 95% CI: 1.10–1.50; model 3: OR = 1.40, 95% CI: 1.03–1.90).

**Table 2:** Associations of serum oleic acid with depression (*n* = 4,382)

	Crude model		Model 1^a^		Model 2^b^		Model 3^c^	
	OR (95% CI)	*p*-value	OR (95% CI)	*p*-value	OR (95% CI)	*p*-value	OR (95% CI)	*p*-value
Per 1 mmol/L increase	1.35 (1.16, 1.57)	0.003	1.40 (1.21, 1.61)	< 0.001	1.29 (1.10, 1.50)	0.003	1.40 (1.03, 1.90)	0.034
Quartiles								
Q1 (≤ 1.53 mmol/L)	Reference (1)		Reference (1)		Reference (1)		Reference (1)	
Q2 (> 1.53 to 1.93 mmol/L)	1.22 (0.82, 1.82)	0.319	1.25 (0.82, 1.90)	0.262	1.31 (0.83, 2.06)	0.216	1.37 (0.82, 2.28)	0.197
Q3 (> 1.93 to < 2.47 mmol/L)	1.30 (0.93, 1.83)	0.124	1.33 (0.91, 1.95)	0.135	1.37 (0.86, 2.21)	0.169	1.51 (0.86, 2.67)	0.122
Q4 (≥ 2.47 mmol/L)	2.03 (1.38, 3.00)	< 0.001	2.21 (1.60, 3.23)	< 0.001	1.93 (1.23, 3.04)	0.008	2.22 (1.04, 4.73)	0.017
*p* for trend	< 0.001		< 0.001		0.009		0.043	

*Abbreviations*: CI Confidence interval, *OR* Odds ratio, *Q* Quantile

In multivariate regression, samples with missing values for covariates in the model were removed

^a^Crude Model: Unadjusted (*n* = 4,382)

^b^Model 1: Adjust for age, sex, and race/ethnicity (*n* = 4,382)

^c^Model 2: Adjust for the variables in Model 1 plus education level, marital status, physical activity, body mass index, smoking status, and alcohol status (*n* = 4,093)

^d^Model 3: Adjust for the variables in Model 2 plus metabolic syndrome, omega-3 polyunsaturated fatty acids, and total cholesterol (*n* = 3,928)

We also converted oleic acid from a continuous variable to a categorical variable (quartiles). In the fully adjusted Model 3, compared with participants in the first oleic acid quartile, participants in the fourth quartile were associated with an increased prevalence of depression (OR = 2.22, 95% CI: 1.04–4.73). Moreover, the *p* value for trend was significant in all models. We also performed sensitivity analyses using datasets containing outliers, and the results were stable (Table S4).

Sensitivity analyses using the five data sets generated by multiple imputations for missing covariates yielded results consistent with those of the primary analysis (Table S5). All covariable subgroup analyses revealed a pattern similar to the main analysis pattern (all *p* for interaction > 0.05) (Table S6).

According to a recent study, a PHQ-9 cutoff score of 10 may overestimate the prevalence of depression [35]. Moreover, a systematic review reports that the overall sensitivity (0.37–0.98) and selectivity (0.42–0.99) of PHQ-9 to be ranged widely in primary care samples [36]. Therefore, we also examined the correlation between serum oleic acid levels and PHQ-9 scores as a sensitivity analysis. Because of the approximate gamma distribution of the PHQ-9 score, the association of oleic acid with the PHQ-9 score was tested by generalized linear models (log-link function and a gamma distribution). Because the outcome variable could not include 0 in the model, an increase of 0.1 in the original PHQ-9 score was used as the outcome variable (0.1–27.1). We also analyzed the association between oleic acid levels and PHQ-9 score using linear regression. Analyses of the PHQ-9 score as a continuous variable were performed as follows. We found that serum oleic acid levels were positively correlated with PHQ-9 score (Table S7).

## Discussion

We examined the association between serum oleic acid levels and depression in this population-based cross-sectional study. Higher serum oleic acid levels were associated with a higher prevalence of depression in the included American population after adjusting for potential confounders.

Previous studies have examined the association between oleic acid levels and MDD but have yielded inconsistent results. In a study of 134 participants aged 6–18 years by Zhou et al., plasma oleic acid levels were lower in both 52 first-episode drug-naive patients with MDD and 32 drug-treated patients with MDD than in the healthy controls [8]. Ding et al. collected plasma samples from 25 healthy controls and 46 patients with MDD, including 23 patients with previous early life stress and 23 patients without early life stress, and found a decrease in oleic acid in patients with MDD than in the healthy controls [9]. McNamara et al. studied the FA composition of the cadaveric superior temporal gyrus and found no significant differences in oleic acid levels between patients with MDD and healthy controls [10].

However, in a case–control study of 137 patients with recurrent MDD and 65 matched non-depressive controls, plasma oleic acid levels were significantly higher in patients with recurrent MDD than in the healthy controls [11]. Using a genetic instrumental variable design, Zeng et al. found oleic acid to be associated with depression risk [37]. These studies showed inconsistent results, which may be due to the sample sizes and different study populations. Studies that used small sample sizes and did not control for confounding factors found reduced or unchanged oleic acid levels in patients with MDD. Our research on the relationship between oleic acid levels and depression used a sample pool of adults with marked racial/ethnic diversity from NHANES. To the best of our knowledge, this observational study used the largest sample size to examine the association between oleic acid levels and depression.

This study adjusted for confounding variables in the analysis of the relationship between serum oleic acid levels and depression. Confounding variables were selected for this study based on previous literature [11, 27]. Age, sex, and race/ethnicity were chosen as covariates because they are basic demographic information and have been shown to be associated with lipid metabolism and depression [38]. Education level and marital status were chosen as covariates because they are sociological factors that are commonly adjusted for when exploring the relationship between depression and lipid metabolism [39–41]. These variables can influence an individual's lifestyle, social support, and access to healthcare resources, which could affect the depressive conditions. Body mass index, smoking status, alcohol status, and physical activity were included because lifestyle habits and health status are known to impact both lipid levels and depression. Metabolic syndrome, omega-3 polyunsaturated fatty acids, and total cholesterol all reflect lipid metabolism and have all been shown to be associated with depression [42–44]. Adjusting for these confounding factors can provide a better understanding of the relationship between serum oleic acid levels and depression.

Several mechanisms may underlie the relationship between oleic acid levels and depression. Oleic acid can reduce the expression of neuropeptide Y in the hypothalamus [45], which has an antidepressant-like effects and plays a major role in stress responses and resilience. In human neural cells, excess oleic acid could cause α-synuclein inclusion formation, resulting in neurotoxicity [46]. Locally increased oleic acid levels at the ependymal surface of the brain could result in the inhibition of neural stem cell proliferation and the deterioration of neurogenic niches [47]. Additionally, oleic acid promotes lipid accumulation and induces an inflammatory phagocyte phenotype [14]. In summary, the mechanisms underlying the oleic acid-depression relationship may be related to neuroinflammation, neurotransmitter disorders, and nerve cell damage. These are the possible mechanisms, as a cross-sectional analysis based on the NHANES database, we were unable to validate the above mechanisms. We will explore the mechanisms underlying the oleic acid-depression relationship in future studies (e.g., exploring the role that resilience plays).

A clearer understanding of the role of oleic acid in depression may lead to new preventive and therapeutic methods. The blood oleic acid concentration reflects the mixed effects of endogenous processing and recent consumption [48]. The endogenous synthesis of oleic acid depends on a rate-limiting step catalyzed by stearoyl-CoA desaturase (SCD), and the concentration of oleic acid could be reduced by SCD inhibitors [7]. Studies have shown that SCD inhibitors play a beneficial role by reducing the oleic acid concentration in animal models of Parkinson's disease [46], Alzheimer's disease [47], and demyelinating disorders [14]. Additionally, oleic acid intake is positively associated with symptoms of anxiety and depression in women [12, 13]. A high-fat diet can increase oleic acid levels and promote depression-like behavior in mice, and the serum oleic acid level in mice significantly correlates with depressive-like behavior [49]. It is necessary to further investigate the potential significance of changing circulating oleic acid levels in depression.

Our study had a few limitations. Owing to the cross-sectional study design of the NHANES, no causal links between serum oleic acid and depression could be established. Second, our community population was not a population of patients with clinically diagnosed MDD, highlighting the need for similar large studies of patients with MDD in the future to fully generalize our findings to these patients.

## Conclusion

Our research, using a cohort of adults with marked racial/ethnic diversity from the NHANES, suggested a positive association between serum oleic acid levels and depression. A better understanding of the role of oleic acid in depression may lead to new preventive and therapeutic methods. Thus, carefully designed prospective studies are necessary to explore the positive effects of changing serum oleic acid levels through diet, medicine, or other measures on depression.

### Supplementary Information

**Additional file 1: Fig. S1.** Flowchart for inclusion of study participants. **Table S1.** Confounding variable list. **Table S2.** Characteristics of the included and excluded populations. **Table S3.** Missing covariates of study participants (*n*=4,459). **Table S4.** Associations of serum oleic acid with depression (Outliers included: *n* = 4,459). **Table S5.** Associations of serum oleic acid with depression after adjusting for covariates that used multiple imputations to handle missing values (*n* = 4382). **Table S6.** Subgroup analysis of the effect of oleic acid on depression. **Table S7.** Associations of serum oleic acid with PHQ-9 score (*n* = 4382).

## Data Availability

The data used in this study are available on the National Health and Nutrition Examination Survey website: https://www.cdc.gov/nchs/nhanes/index.htm.

## Abbreviations

FA: Fatty acid
MUFA: Monounsaturated fatty acid
MDD: Major depressive disorder
NHANES: National Health and Nutrition Examination Survey
CDC: Centers for Disease Control and Prevention
PHQ-9: Patient Health Questionnaire-9
BMI: Body mass index
SCD: Stearoyl-CoA desaturase
MetS: Metabolic syndrome
