# Supplementary material for: Association of serum oleic acid level with depression in American adults: a cross-sectional study
Source: BMC Psychiatry. 2023 Nov 16;23:845. doi: 10.1186/s12888-023-05271-0 (PMC10652490; doi:10.1186/s12888-023-05271-0)
Supplement: Supplementary file 1 — Additional file 1: Fig. S1. Flowchart for inclusion of study participants. Table S1. Confounding variable list. Table S2. Characteristics of the included and excluded populations. Table S3. Missing covariates of study participants (n=4,459). Table S4. Associations of serum oleic acid with depression (Outliers included: n = 4,459). Table S5. Associations of serum oleic acid with depression after adjusting for covariates that used multiple imputations to handle missing values (n = 4382). Table S6. Subgroup analysis of the effect of oleic acid on depression. Table S7. Associations of serum oleic acid with PHQ-9 score (n = 4382). [file 12888_2023_5271_MOESM1_ESM.docx]

**Supplementary material**

Excluded participants <18 years old (*n*=7,954)

Excluded participants without fatty acid test results or fasting laboratory specimens (*n*=7020)

(*n*=3245)

Excluded participants with incomplete depression questionnaire (*n*=452)

Excluded participants with absence of oleic acid data (*n*=46)

4,459 participants were included in the study

Total subjects in NHANES

2011–2014 (*n*=19,931)

Fig. S1. Flowchart for inclusion of study participants

Abbreviation: NHANES, National Health and Nutrition Examination Survey

Table S1. Confounding variable list

| Covariate | | Detailed definition | |
| --- | --- | --- | --- |
| Categorical variable | | | |
| Sex | Male | | |
|  | Female | | |
| Race/ethnicity | Non-Hispanic white | | |
|  | Non-Hispanic black | | |
|  | Mexican American | | |
|  | Other Hispanic | | |
|  | Other race/multiple races | | |
| Education level | <High school | | |
|  | Completed high school | | |
|  | >High school | | |
| Marital status | Married/Living with partner | | |
|  | Widowed/Divorced/Separated/Never married | | |
| Alcohol status | Yes | | Determined by answers to the question, “In any one year, have you had at least 12 drinks of any type of alcoholic beverage?” |
|  | No | |  |
| Smoking status | Never smoking | | Smoked <100 cigarettes. |
|  | Former smoker | | Not currently smoking but smoked ≥100 cigarettes. |
|  | Current smoker | | ≥100 cigarettes and currently smoking every day or some days. |
| Physical activity | Inactive | | Reporting no moderate activity or vigorous activity. |
|  | Moderate | | Moderate physical activity (e.g., brisk walking, swimming, bicycling at a regular pace) reported. |
|  | Vigorous | | High-intensity activities, fitness, and sports such as running or basketball reported. |
|  | Both moderate and vigorous | | Both moderate and vigorous activity reported. |
| Metabolic Syndrome | Yes | | Defined according to the updated National Cholesterol Education Program/Adult Treatment Panel III criteria for Americans. |
|  | No | |  |
| Body mass index | <25.0 kg/m2 | | Weight (kg) divided by height (m) squared. |
|  | 25.0 to <30.0 kg/m2 | |  |
|  | ≥30.0 kg/m2 | |  |
| Continuous variable | | | |
| Age (years) | - | | |
| Total cholesterol (mmol/L) | Measured by single reagent enzymatic method, an endpoint reaction specific for cholesterol. Detailed laboratory test information is available at: https://wwwn.cdc.gov/Nchs/Nhanes/2011-2012/TCHOL_G.htm | | |
| Omega-3 polyunsaturated fatty acids (μmol/L) | Detected using gas chromatograph together with oleic acid. Detailed laboratory test information is available at: https://wwwn.cdc.gov/nchs/data/nhanes/2011-2012/labmethods/FAS_G_MET.PDF | | |

Table S2. Characteristics of the included and excluded populations

| Characteristic | Excluded | Included | *p*-value |  |
| --- | --- | --- | --- | --- |
|  |  |  |  |  |
|  |  |  |  |  |
| Sample size | 7518 | 4459 |  |  |
| Male, n (%) | 3606 (48.0) | 2207 (49.5) | 0.109 |  |
| Age, years, mean (SD) | 47.29 (18.63) | 47.53 (18.45) | 0.495 |  |
| Educational level, *n* (%) |  |  | 0.03 |  |
| <High school | 1805 (24.0) | 1015 (22.8) |  |  |
| Completed high school | 1716 (22.9) | 964 (21.6) |  |  |
| >High school | 3987 (53.1) | 2478 (55.6) |  |  |
| Race/ethnicity, *n* (%) |  |  | <0.001 |  |
| Non-Hispanic White | 2799 (37.2) | 1880 (42.2) |  |  |
| Non-Hispanic Black | 1861 (24.8) | 948 (21.3) |  |  |
| Mexican American | 899 (12.0) | 539 (12.1) |  |  |
| Other Hispanic | 720 (9.6) | 436 (9.8) |  |  |
| Other race/multiple races | 1239 (16.5) | 656 (14.7) |  |  |
| BMI, n (%) |  |  | 0.182 |  |
| <25.0 kg/m2 | 2304 (33.1) | 1391 (31.5) |  |  |
| 25.0 to <30.0 kg/m2 | 2172 (31.2) | 1423 (32.2) |  |  |
| ≥30.0 kg/m2 | 2478 (35.6) | 1604 (36.3) |  |  |
| Marital status, *n* (%) |  |  | 0.003 |  |
| Married/Living with partner | 4008 (56.4) | 2497 (59.3) |  |  |
| Widowed/Divorced/Separated/Never married | 3100 (43.6) | 1714 (40.7) |  |  |
| Alcohol status, *n* (%) | 4170 (70.3) | 3197 (71.8) | 0.095 |  |
| Smoking status, *n* (%) |  |  | 0.169 |  |
| Never smoking | 4215 (57.5) | 2501 (57.6) |  |  |
| Former smoker | 1606 (21.9) | 1000 (23.0) |  |  |
| Current smoker | 1504 (20.5) | 838 (19.3) |  |  |
| Physical activity, *n* (%) |  |  | 0.175 |  |
| Inactive | 3823 (50.9) | 2178 (48.8) |  |  |
| Moderate | 1970 (26.2) | 1224 (27.5) |  |  |
| Vigorous | 633 (8.4) | 400 (9.0) |  |  |
| Both moderate and vigorous | 1091 (14.5) | 657 (14.7) |  |  |
| Total cholesterol, mmol/L, mean (SD) | 4.91 (1.10) | 4.89 (1.06) | 0.218 |  |
| Omega-3 PUFAs, μmol/L, mean (SD) | 391.12 (189.09) | 370.55 (178.16) | 0.009 |  |
| Metabolic Syndrome, *n* (%) | 1929 (26.0) | 1463 (33.1) | <0.001 |  |

Abbreviations: BMI, body mass index; PUFAs, polyunsaturated fatty acids; SD, standard deviation.

Table S3. Missing covariates of study participants (*n*=4,459)

| Variable | Number of patients (% missing) |
| --- | --- |
| Age | 0 (0%) |
| Sex | 0 (0%) |
| Race/ethnicity | 0 (0%) |
| Education level | 2 (0.04%) |
| Marital status | 248 (5.56%) |
| Alcohol status | 6 (0.13%) |
| Smoking status | 120 (2.69%) |
| Physical activity | 0 (0%) |
| BMI | 41 (0.92%) |
| Total cholesterol | 26 (0.58%) |
| Omega-3 PUFA | 117 (2.62%) |
| Metabolic Syndrome | 38 (0.85%) |

Abbreviations: BMI, body mass index; PUFA, polyunsaturated fatty acid

Table S4. Associations of serum oleic acid with depression (Outliers included: *n* = 4,459).

|  | Crude model^a^ | | Model 1^b^ | | Model 2^c^ | | Model 3^d^ | |
| --- | --- | --- | --- | --- | --- | --- | --- | --- |
|  | OR (95% CI) | *p*-value | OR (95% CI) | *p*-value | OR (95% CI) | *p*-value | OR (95% CI) | *p*-value |
| Per 1 mmol/L increase | 1.14 (1.05, 1.24) | 0.004 | 1.17 (1.08, 1.26) | <0.001 | 1.12 (1.02, 1.23) | 0.024 | 1.23 (1.02, 1.47) | 0.031 |
| Quartiles |  |  |  |  |  |  |  |  |
| Q1 (≤1.54 mmol/L) | Reference (1) |  | Reference (1) |  | Reference (1) |  | Reference (1) |  |
| Q2 (>1.54 to 1.94 mmol/L) | 1.22 (0.84, 1.78) | 0.284 | 1.25 (0.84, 1.85) | 0.261 | 1.29 (0.83, 1.98) | 0.229 | 1.41 (0.84, 2.35) | 0.165 |
| Q3 (>1.94 to <2.51 mmol/L) | 1.29 (0.92, 1.81) | 0.131 | 1.33 (0.91, 1.94) | 0.134 | 1.34 (0.84, 2.14) | 0.19 | 1.53 (0.87, 2.69) | 0.122 |
| Q4 (≥ 2.51 mmol/L) | 2.09 (1.45, 3.00) | <0.001 | 2.27 (1.60, 3.23) | <0.001 | 1.97 (1.30, 2.98) | 0.004 | 2.52 (1.23, 5.17) | 0.017 |
| *p* for trend | <0.001 |  | <0.001 |  | 0.005 |  | 0.018 |  |

Abbreviations: CI, confidence interval; OR, odds ratio; Q, quantile.

In multivariate regression, samples with missing values for covariates in the model were removed.

^a^Crude Model: Unadjusted (*n* = 4,459).

^b^Model 1: Adjust for age, sex, and race/ethnicity (*n* = 4,459).

^c^Model 2: Adjust for the variables in Model 1 plus education level, marital status, physical activity, body mass index, smoking status, and alcohol status (*n* = 4,169).

^d^Model 3: Adjust for the variables in Model 2 plus metabolic syndrome, omega-3 polyunsaturated fatty acids, and total cholesterol (*n* = 4,003).

Table S5. Associations of serum oleic acid with depression after adjusting for covariates that used multiple imputations to handle missing values (n = 4382).

| Groups | Dataset 1 | | Dataset 2 | | Dataset 3 | | Dataset 4 | | Dataset 5 | |
| --- | --- | --- | --- | --- | --- | --- | --- | --- | --- | --- |
|  | OR (95% CI) | *p*-value | OR (95% CI) | *p*-value | OR (95% CI) | *p*-value | OR (95% CI) | *p*-value | OR (95% CI) | *p*-value |
| Per 1 mmol/L increase | 1.38 (1.03, 1.84) | 0.033 | 1.39 (1.03, 1.86) | 0.032 | 1.38 (1.03, 1.85) | 0.032 | 1.38 (1.03, 1.85) | 0.033 | 1.38 (1.03, 1.85) | 0.033 |
| Quartiles |  |  |  |  |  |  |  |  |  |  |
| Q1 (≤1.54 mmol/L) | Reference (1) |  | Reference (1) |  | Reference (1) |  | Reference (1) |  | Reference (1) |  |
| Q2 (>1.54 to 1.94 mmol/L) | 1.31 (0.84, 2.06) | 0.206 | 1.32 (0.84, 2.08) | 0.199 | 1.31 (0.83, 2.07) | 0.209 | 1.31 (0.83, 2.05) | 0.211 | 1.31 (0.83, 2.05) | 0.21 |
| Q3 (>1.94 to <2.505 mmol/L) | 1.40 (0.85, 2.31) | 0.159 | 1.41 (0.85, 2.32) | 0.156 | 1.41 (0.85, 2.33) | 0.156 | 1.39 (0.85, 2.29) | 0.165 | 1.40 (0.85, 2.30) | 0.157 |
| Q4 (≥ 2.505 mmol/L) | 2.39 (1.22, 4.68) | 0.017 | 2.41 (1.22, 4.74) | 0.017 | 2.40 (1.22, 4.73) | 0.017 | 2.38 (1.21, 4.65) | 0.017 | 2.39 (1.22, 4.69) | 0.017 |
| *p* for trend | 0.02 | | 0.02 | | 0.019 | | 0.02 | | 0.02 | |

Abbreviations: CI, confidence interval; OR, odds ratio; Q, quantile.

The missForest package was used to impute missing data for study participants. This package is a random forest-based technique that is highly computationally efficient for high-dimensional data consisting of both categorical and continuous predictors. Five imputed datasets were generated based on the missForest package. All covariates with missing values were inputted, including education level, marital status, body mass index, smoking status, alcohol status, metabolic syndrome, omega-3 polyunsaturated fatty acids, and total cholesterol.

Table S6. Subgroup analysis of the effect of oleic acid on depression.

| Subgroup | Number of participants | OR (95% CI) | *p* for interaction |
| --- | --- | --- | --- |
| Overall |  |  |  |
| Crude | 4382 | 1.35 (1.16, 1.57) |  |
| Adjusted | 3928 | 1.40 (1.03, 1.90) |  |
| Sex |  |  | 0.604 |
| Female | 1988 | 1.24 (0.88, 1.75) |  |
| Male | 1940 | 1.69 (1.12, 2.56) |  |
| Age (years) |  |  | 0.365 |
| ≥18, <45 | 1669 | 1.80 (1.07, 3.02) |  |
| ≥45, <60 | 963 | 1.52 (0.95, 2.42) |  |
| ≥60 | 1296 | 1.19 (0.74, 1.92) |  |
| Race/ethnicity |  |  | 0.364 |
| Non-Hispanic White | 1688 | 1.50 (0.95,2.37) |  |
| Non-Hispanic Black | 804 | 1.35 (0.87, 2.08) |  |
| Mexican American | 464 | 0.69 (0.18, 2.61) |  |
| Other Hispanic | 389 | 1.97 (0.75, 5.20) |  |
| Other race/multiple races | 583 | 0.77 (0.39, 1.53) |  |
| Educational level |  |  | 0.819 |
| <High school | 847 | 1.29 (0.78, 2.12) |  |
| Completed high school | 828 | 1.32 (0.71, 2.46) |  |
| >High school | 2253 | 1.44 (1.00, 2.06) |  |
| Physical activity |  |  | 0.521 |
| Inactive | 1923 | 1.57 (1.03, 2.40) |  |
| Moderate | 1108 | 1.04 (0.66, 1.65) |  |
| Vigorous | 331 | 1.20 (0.14, 10.60) |  |
| Both moderate and vigorous | 566 | 1.23 (0.40, 3.79) |  |
| BMI category |  |  | 0.466 |
| <25.0 kg/m^2^ | 1201 | 1.46 (0.81, 2.62) |  |
| 25.0 to <30.0 kg/m^2^ | 1273 | 1.81 (0.81, 3.91) |  |
| ≥30.0 kg/m^2^ | 1454 | 1.19 (0.82, 1.71) |  |
| Marital status |  |  | 0.833 |
| Married/Living with partner | 2338 | 1.26 (0.77, 2.04) |  |
| Widowed/Divorced/Separated/Never married | 1590 | 1.59 (1.17, 2.16) |  |
| Smoking status |  |  | 0.771 |
| Never smoker | 2235 | 1.30 (0.85, 2.00) |  |
| Former smoker | 931 | 1.38 (0.70, 2.72) |  |
| Current smoker | 762 | 1.48 (0.84, 2.62) |  |
| Alcohol status |  |  | 0.219 |
| No | 1055 | 1.02 (0.49, 2.11) |  |
| Yes | 2873 | 1.59 (1.11, 2.27) |  |
| Metabolic Syndrome |  |  | 0.139 |
| No | 2598 | 2.11 (1.27, 3.50) |  |
| Yes | 1330 | 1.17 (0.83, 1.65) |  |
| Omega-3 polyunsaturated fatty acids |  |  | 0.454 |
| Low | 1311 | 1.46 (0.66,3.23) |  |
| Middle | 1308 | 1.80 (1.06, 3.06) |  |
| High | 1309 | 1.19 (0.85, 1.67) |  |
| Total cholesterol |  |  | 0.895 |
| Low | 1342 | 1.31 (0.79, 2.17) |  |
| Middle | 1283 | 1.33 (0.76, 2.33) |  |
| High | 1303 | 1.55 (1.01, 2.36) |  |

Abbreviations: CI, confidence interval; OR, odds ratio.

Subgroup analyses were performed using all covariates in Table S1. Analyses were adjusted for all covariates. Samples with missing values for covariates were removed. Stratified variables were not adjusted in the corresponding model.

Table S7. Associations of serum oleic acid with PHQ-9 score (*n* = 4382).

|  | Model 1 | | Model 2 | | Model 3 | | Model 4 | |
| --- | --- | --- | --- | --- | --- | --- | --- | --- |
|  | β (95% CI) | *p*-value | β (95% CI) | *p*-value | β (95% CI) | *p*-value | β (95% CI) | *p*-value |
| Per 1 mmol/L increase | 0.13 (0.06, 0.20) | <0.001 | 0.14 (0.01, 0.27) | 0.043 | 0.42 (0.19, 0.65) | <0.001 | 0.45 (0.02, 0.88) | 0.042 |
| Quartiles |  |  |  |  |  |  |  |  |
| Q1 (≤1.53 mmol/L) | Reference (0) |  | Reference (0) |  | Reference (0) |  | Reference (0) |  |
| Q2 (>1.53 to 1.93 mmol/L) | -0.01 (-0.18, 0.15) | 0.862 | 0.01 (-0.27, 0.28) | 0.947 | -0.04 (-0.50, 0.42) | 0.862 | 0.06 (-0.71, 0.84) | 0.812 |
| Q3 (>1.93 to <2.47 mmol/L) | 0.11 (-0.02, 0.25) | 0.094 | 0.19 (-0.10, 0.48) | 0.132 | 0.33 (-0.06, 0.72) | 0.09 | 0.52 (-0.25, 1.29) | 0.12 |
| Q4 (≥ 2.47 mmol/L) | 0.26 (0.08, 0.44) | 0.006 | 0.29 (-0.11, 0.70) | 0.106 | 0.82 (0.24, 1.39) | 0.007 | 0.90 (-0.27, 2.06) | 0.092 |
| *p* for trend | 0.001 |  | 0.042 |  | 0.002 |  | 0.037 |  |

Abbreviations: CI, confidence interval; Q, quantile; PHQ-9, Patient Health Questionnaire-9.

In multivariate regression, samples with missing values for covariates in the model were removed. An increase of 0.1 in the original PHQ-9 score was used as the outcome variable (0.1-27.1) in models 1 and 2. The original PHQ-9 score was used as the outcome variable (0-27) in models 3 and 4. Models 1 and 2 used generalized linear models with a log-link function and a gamma distribution. Models 3 and 4 used linear regression models. Models 1 and 3 were unadjusted (n = 4382). Models 2 and 4 were adjusted for age, sex, race/ethnicity, education level, marital status, physical activity, body mass index, smoking status, alcohol status, metabolic syndrome, omega-3 polyunsaturated fatty acids, and total cholesterol (n = 3928).
